# Microelimination of Hepatitis C in Thailand, Phetchabun Model: Progress, Challenges, and Future Directions

**DOI:** 10.3390/jcm14113946

**Published:** 2025-06-03

**Authors:** Yong Poovorawan, Sitthichai Kanokudom, Nungruthai Suntronwong, Pornjarim Nilyanimit, Ritthideach Yorsaeng, Wijittra Phaengkha, Napaporn Pimsing, Chatree Jullapetch

**Affiliations:** 1 Center of Excellence in Clinical Virology, Faculty of Medicine, Chulalongkorn University, Bangkok 10330, Thailand; sitthichai.k@chula.ac.th (S.K.); nungruthai.s@chula.ac.th (N.S.); mim_bhni@hotmail.com (P.N.); ritthideach.yor@gmail.com (R.Y.); 2 Fellow of the Royal Society of Thailand (FRS [T]), Royal Society of Thailand, Sanam Sueapa, Dusit, Bangkok 10300, Thailand; 3 Nam Nao Hospital, Nam Nao District, Phetchabun 67260, Thailand; dr.zontok12@gmail.com; 4 Phetchabun Provincial Public Health Office, Phetchabun 67000, Thailand; napaporn.tu16@gmail.com; 5 Division of AIDS and STIs, Department of Disease Control, Ministry of Public Health, Nonthaburi 11000, Thailand; chai6127@hotmail.com

**Keywords:** hepatitis C, epidemiology, public health, genotype, microelimination, Phetchabun, Thailand, model, challenges, future direction

## Abstract

Hepatitis C virus (HCV) remains a global health challenge, contributing to chronic liver disease and hepatocellular carcinoma. In Thailand, HCV prevalence has declined from ~2% in the 1990s due to universal blood screening, harm reduction, and expanded treatment. This narrative review draws on diverse sources—including PubMed and Scopus databases, international and national health websites, government reports, and local communications—to compile epidemiological data, genotype distribution, and elimination strategies, with a focus on Phetchabun province, Thailand, as a model for achieving the World Health Organization’s (WHO) hepatitis C elimination targets. National surveys in 2004, 2014, and 2024 show a prevalence drop from 2.15% to 0.56%. However, HCV persists among high-risk groups, including people who inject drugs, people living with HIV, patients undergoing maintenance hemodialysis, and prisoners. Thailand’s National Health Security Office has expanded treatment access, including universal screening for those born before 1992. The Phetchabun Model, launched in 2017, employs a decentralized test-to-treat strategy. By April 2024, 88.64% (288,203/324,916) of the target population was screened, and 4.88% were anti-HCV positive. Among those tested, 72.61% were HCV-RNA positive, and 88.17% received direct-acting antivirals (i.e., SOF/VEL), achieving >96% sustained virological response. The Phetchabun Model demonstrates a scalable approach for HCV elimination. Addressing testing costs, improving access, and integrating microelimination strategies into national policy are essential to achieving the WHO’s 2030 goals.

## 1. Introduction

Hepatitis C virus (HCV) remains a significant global health burden, leading to chronic hepatitis, liver cirrhosis, and hepatocellular carcinoma (HCC). Over 50 million people worldwide live with chronic HCV infection, with approximately 1 million new cases occurring each year. According to the World Health Organization (WHO), an estimated 242,000 people died from hepatitis C in 2022, mostly due to cirrhosis and HCC (primary liver cancer) [1]. The prevalence of HCV varies widely across regions. The highest burden is observed in low- and middle-income countries. Notably, Egypt previously had one of the highest prevalence rates globally; however, aggressive national treatment campaigns have significantly reduced it [2,3]. Other countries with high prevalence include Pakistan, where estimates suggest around 5–7% of the population is infected [4,5,6,7], and regions in sub-Saharan Africa [8,9] and Central and East Asia [10,11], where unsafe medical practices and limited access to diagnosis and treatment continue to drive transmission. In Thailand, the prevalence of anti-HCV among new blood donors was approximately 2% in the 1990s [12]. This suggests that over one million people in the country may have been infected. HCV has played a significant role in liver disease and related mortality in Thailand.

Efforts to control the spread of HCV in Thailand can help reduce its prevalence. Key interventions in the prevention and control of viral transmission include public health measures implemented over the years. In 1984, the first case of human immunodeficiency virus (HIV) infection was identified in Thailand, and the number of cases increased significantly among people who inject drugs (PWID) from the mid-1990s onward, as well as among sex workers and their clients [13]. This situation led to the implementation of universal precautions and mandatory blood donor screening, which has been in place since 1992 [12]. In 2004, additional harm reduction programs were introduced to support PWID. These initiatives included access to sterile syringes, education on HIV and HCV transmission, safer injection techniques, and drug use awareness. Access to sterile syringes remains a considerable challenge. It is crucial to raise awareness of its importance, and various organizations must work together to promote harm reduction [14]. These measures have contributed to a steady decline in HIV and HCV incidence, as confirmed by mathematical models predicting further reductions. However, complete elimination remains a challenge [15].

Recognizing the feasibility of HCV elimination, the 2015 Glasgow Declaration set the first global target to eliminate viral hepatitis as a public health threat by 2030 [16], a goal later adopted by the WHO in 2016 [17]. The outlined targets for hepatitis C elimination include reducing new HCV infections by at least 90%, ensuring that at least 90% of the population knows their HCV status through screening, and treating at least 80% of diagnosed individuals. The ultimate goal is to decrease HCV-related mortality by at least 65% (Table 1). Thailand embraced this commitment but faced early implementation delays, further complicated by the COVID-19 pandemic.

This narrative review draws on our extensive experience in hepatitis research in Thailand, with a focus on the epidemiology of HCV, historical trends, current control strategies, and the microelimination model implemented in Phetchabun province (a high prevalence area). Initiated in 2017, the Phetchabun Model is a pioneering effort in HCV elimination, offering valuable insights for national scale-up and serving as a potential model for other countries with similar epidemiological and socioeconomic contexts. The aim is to share lessons learned and provide a practical framework to support the World Health Organization’s goal of eliminating viral hepatitis as a public health threat by 2030.

## 2. Narrative Review Methodology

We conducted a comprehensive literature review to gather relevant articles related to hepatitis C, with a particular focus on the Thai context. Data were sourced from PubMed, Scopus, Google, Google Scholar, international and national health organization websites, local communications, and other relevant databases using multiple keyword combinations, such as “hepatitis”, “hepatitis C”, “HCV”, “epidemiology”, “serology”, “elimination”, “guideline”, “treatment”, “control”, “antiviral agents”, “WHO”, “in Phetchabun”, “in Thailand”, “in Egypt”, and other related terms. All studies conducted from 1980 to 2025 were screened. Studies that included participants of all age groups, statuses, and characteristics were considered.

Two investigators (Yong Poovorawan and Sitthichai Kanokudom) independently screened the titles, abstracts, and full-text articles. Initially, only titles and abstracts were reviewed. Subsequently, the full-text papers of potentially relevant studies were thoroughly examined. Studies written in Thai and derived from local communications were also included. Furthermore, if two studies used the same data source and content, the one with less information or lacking originality was excluded. Any disagreements during the selection process were resolved through consensus among at least two authors.

## 3. Epidemiology of Hepatitis C in Thailand

HCV was first recognized in Thailand over 40 years ago. At that time, anti-HCV prevalence was about 2.6% in healthy blood donors and 2.8% in healthy pregnant women [18]. A few years later, the prevalence in the general population was approximately 2.0% [19]. The prevalence was even higher among high-risk groups, including PWID [20], individuals who regularly received blood transfusions or blood products [21], and hemodialysis patients [22]. Following this, various preventive measures were implemented, such as universal blood screening for all donated blood and blood products starting in 1992 [12]. Additionally, increasingly sensitive detection methods were introduced, including the use of high-sensitivity enzyme-linked immunosorbent assay (ELISA), which continued to improve over time. Nucleic acid testing (NAT) was later added in 2006 to enhance detection accuracy [12].

**Figure 1:**
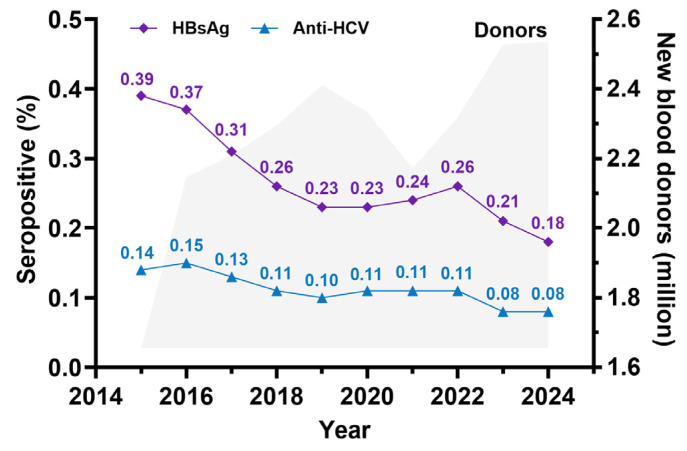
Seropositivity rate of HBsAg and anti-HCV derived from new blood donor screening by the Thailand National Blood Center (NBC) and the regional blood center between 2015 and 2024. (The figure was established based on data from NBC, the Thailand Red Cross Society).

With the emergence of the HIV epidemic, further preventive measures were established, particularly targeting shared needle use and the implementation of universal precautions in healthcare settings. Subsequently, increased awareness of bloodborne diseases, such as HIV, hepatitis B, hepatitis C, and syphilis, has led to greater harm reduction efforts, increased condom use, and more frequent personal blood testing. These interventions have contributed to a continuous decline in the incidence of HCV infection in Thailand. This trend is evident in blood donor screening, where the detection rate of HCV in new blood donors has significantly decreased, as illustrated in Figure 1.

A national seroprevalence survey was conducted in Thailand to assess HCV infection in the general population using anti-HCV testing in the years 2004, 2014, and 2024 [23,24]. The surveys included individuals aged 6 months to 60 years, except for the 2024 survey, which extended the age range to 80 years. The findings revealed that HCV prevalence was higher among adults. The overall anti-HCV positivity rates in the general population declined from 2.15% in 2004 [23] to 0.94% in 2014 [24] and subsequently to 0.56% in 2024. Among those who tested positive for anti-HCV, approximately 72.4% were found to have detectable HCV RNA [24]. These findings indicate a continuous decline in HCV infection in Thailand.

Our previous study, conducted in 1999, focused on a generally healthy population, particularly blood donors, where HCV seroprevalence was 3.2% [20]. In contrast, HCV prevalence remains high among high-risk groups, including people living with HIV (PLHIV), people who inject drugs (PWID), patients undergoing maintenance hemodialysis (MHD), and prison inmates. Previous data also indicated that HCV prevalence among PWID in Thailand reached 90–92.5% in 1999–2002 [20]. A study by Goel A. and colleagues [25], analyzing HCV seroprevalence data from the published data between 1991 and 2020, reported prevalence rates of 13.45% in PLHIV, 58.86% in PWID, 13.13% in MHD patients, and 5.22% in prison inmates. Additionally, other at-risk populations, including individuals engaging in high-risk sexual behaviors and men who have sex with men (MSM), had HCV prevalence rates of 2.0% and 1.62%, respectively. Based on these prevalence rates and the estimated population sizes of each high-risk group [25], combined with HCV infection in the general population, which is estimated to be 0.56%, it is estimated that 506,387 individuals in Thailand have ever been infected with HCV (detectable anti-HCV), as shown in Table 2.

## 4. Hepatitis C Genotype in Thailand

**Figure 2:**
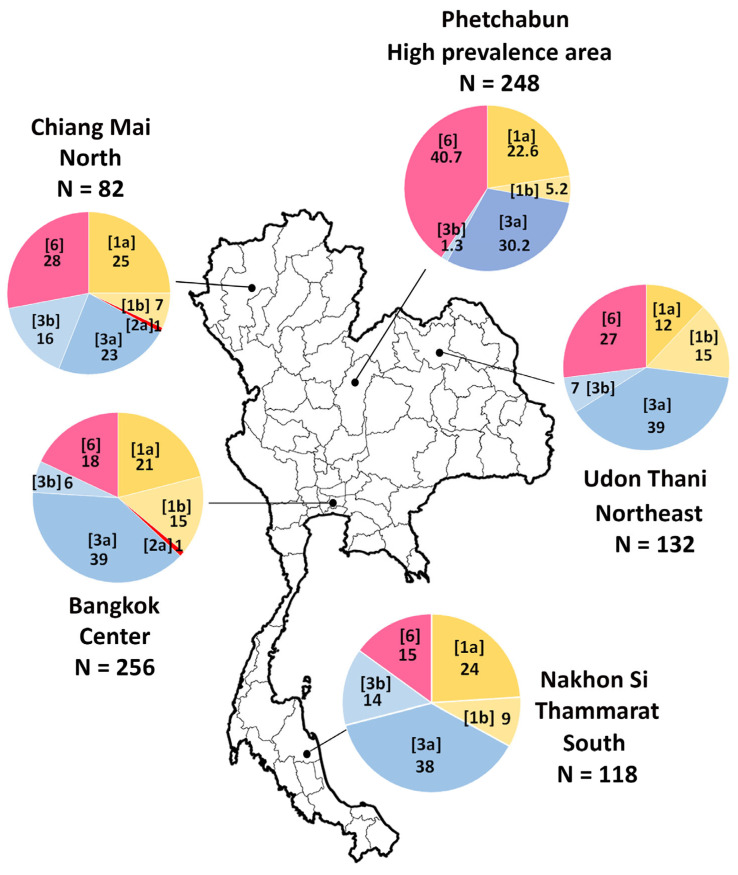
Distribution of HCV genotypes across different regions of Thailand. The study area includes Bangkok, Chiang Mai, Udon Thani, Nakhon Si Thammarat, and Phetchabun provinces. HCV subtypes are presented in the brackets, with numbers representing the percentage of prevalence of each genotype among positive samples. The figure is based on data from references [26,27].

A study conducted between 2007 and 2012 involving 588 individuals identified HCV genotypes 1, 3, and 6 as the predominant genotypes in Thailand, with varying regional distributions. The most detected subtypes were 3a, 1a, 1b, and 6 [26]. However, genotype distribution varied across different geographic regions. In central and southern Thailand, genotype 1a was more prevalent than 1b. In contrast, genotype 6 was the most dominant in northern and northeastern Thailand, particularly in high-endemic areas of the lower northern region [26]. A subsequent study conducted in 2018 in Phetchabun province (n = 248 cases), where the incidence of HCV infection was notably high, confirmed that genotype 6 exhibited significant genetic diversity. Among the genotype 6 subtypes, 6f was the most frequently detected (34.4%), followed by 6n (4%) and 6i (2%) [27]. A detailed illustration of the distribution of HCV genotypes in Thailand is presented in Figure 2.

## 5. Hepatitis C Treatment Policy in Thailand

**Figure 3:**
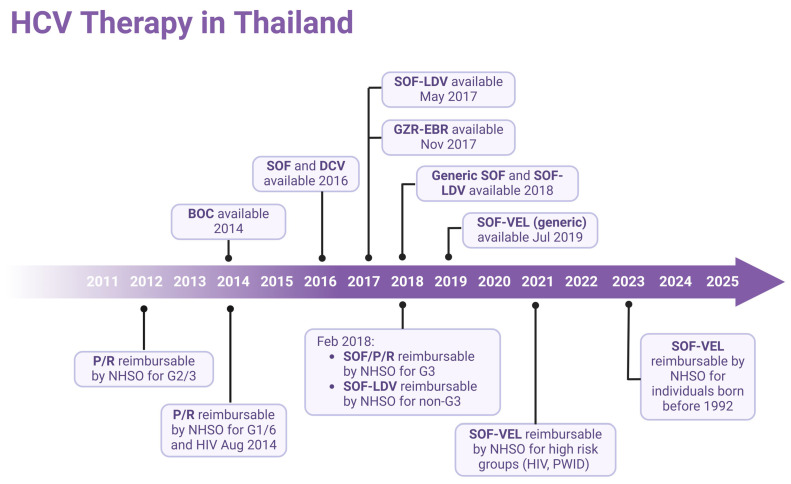
Timeline of antiviral drug availability for HCV and treatment approaches in Thailand. P/R, Peg-Interferon/Ribavirin; BOC, boceprevir; SOF, Sofosbuvir; LDV, Ledipasvir; DCV, daclatasvir; GZR, grazoprevir; EBR, elbasvir; VEL, Velpatasvir; NHSO, National Health Security Office; G, HCV genotype. (Created with BioRender.com).

Over the past years, the treatment of hepatitis C has faced numerous limitations. Initially, treatment efficacy was moderate with Pegylated Interferon (Peg-IFN), which was also restricted to certain genotypes. Additionally, treatment was associated with significant side effects, and highly effective medications were expensive. In the year 2011, the National Health Security Office (NHSO) approved the treatment of hepatitis C using Peg-IFN in combination with Ribavirin (RBV) for patients infected with genotype 3 [28]. Later, in 2014, treatment eligibility was expanded to include genotypes 1 and 6, as well as HIV co-infected patients. By 2018, NHSO updated its treatment protocol, introducing Sofosbuvir (SOF) + Peg-IFN + RBV for genotype 3 and Sofosbuvir/Ledipasvir (SOF/LDV) for non-genotype 3 patients [28]. Following the availability of generic Sofosbuvir/Velpatasvir (SOF/VEL) in 2021, the treatment policy was revised to include high-risk groups, such as PLHIV and PWID [29]. Finally, in 2023, the NHSO approved universal screening and treatment for high-risk groups, including PWID, PLHIV, prison inmates, MSM, and healthcare workers, as well as the general population born before 1992. This significantly improved access to hepatitis B and C care in Thailand [30], as illustrated in Figure 3.

## 6. Eliminating Hepatitis C in Egypt

Egypt once had one of the highest hepatitis C prevalence rates worldwide, with an estimated 15–20% of the general population affected, primarily due to past universal schistosomiasis treatment campaigns in the 1950s to 1980s involving syringe reuse [31,32]. This resulted in a significant burden on the healthcare system, with high rates of liver disease, cirrhosis, and hepatocellular carcinoma. Recognizing the crisis, the Egyptian government launched a national elimination program in 2006, focusing on large-scale screening, cost-effective treatment strategies, and public awareness campaigns [33].

Initial treatment efforts using Peg-IFN and RBV given for 24–48 weeks had limited success, achieving sustained virologic response (SVR) rates in the following guideline criteria of only 45–55%, leaving many patients untreated [34,35]. The introduction of DAAs, particularly Sofosbuvir and Ledipasvir, significantly improved treatment outcomes. However, high drug costs initially limited access. To address this, Egypt negotiated with pharmaceutical companies to lower prices and, in 2016, began producing generic pan-genotypic DAAs at an affordable cost, enabling mass treatment [36].

A key component of the strategy was nationwide screening using WHO-prequalified rapid diagnostic tests (RDTs) or strip tests, followed by confirmatory RT-PCR testing for viremia. This approach allowed for early detection and immediate linkage to treatment. Government commitment, strong international collaboration, and simplified treatment protocols accelerated progress. By 2021, over 4.6 million patients had received treatment, and the prevalence of viremia dramatically decreased from 6% in 2015 to less than 0.5% of the population in 2021 [35]. This success is a landmark achievement in global public health, positioning the country as a model for HCV elimination. This comprehensive approach combining mass screening, affordable and effective treatment, public health awareness, and international support provides a blueprint for other nations striving to meet the WHO’s hepatitis elimination goals by 2030.

## 7. The Phetchabun Model, a Simplified Test-to-Treat Model

A pilot study aimed at eliminating HCV was initiated, selecting Phetchabun province as the pilot site. Phetchabun is a medium-sized province located in lower northern Thailand consisting of 11 districts and situated approximately 346 km north of Bangkok. The province has a total population of 973,386, including 479,134 males and 494,252 females, the majority of whom are engaged in agriculture [37]. This project commenced in 2017, comparing two districts in Phetchabun (Lom Kao and Lom Sak) with the Chum Phae district in the neighboring province of Khon Kaen. Serum samples were collected from approximately 1500 individuals aged 30 to 64 years in each province. The anti-HCV seropositive rates were 15.5% (259/1667) in Phetchabun and 3.6% (51/1410) in Khon Kaen, a nearly fivefold difference [38]. The predominant HCV genotypes identified were genotypes 6, 3, and 1, indicating a high prevalence of HCV in Phetchabun, with major risk factors including intravenous drug use (IVDU) and tattoos [38].

The NHSO established criteria for HCV treatment, including a viral load exceeding 5000 IU/mL and liver fibrosis assessed through FibroScan with a score ≥ 7.5 kPa (or a METAVIR score > F2). Initially, only genotypes 2 and 3 were eligible for treatment with Peg-IFN in 2012, with subsequent modifications in 2014 to include additional genotypes. However, data from Phetchabun indicated that among individuals testing positive for anti-HCV, approximately 80% had detectable HCV-RNA. Furthermore, among those with HCV-RNA positivity, only 78.4% met the NHSO treatment criteria [38]. These stringent eligibility conditions posed significant barriers to HCV screening and treatment accessibility, compounded by the high costs and complexity of diagnostic procedures, such as genotyping, viral load quantification, and liver fibrosis assessment through FibroScan.

A study of younger populations in Phetchabun aged 18 years and above revealed that HCV incidence was significantly higher among individuals born before 1983 (aged ≥ 35 years) [39,40]. This group was identified as a priority for screening to facilitate timely treatment, particularly with pan-genotypic DAAs. Consequently, a pilot project was launched in Phetchabun to simplify the test-to-treat strategy [41]. The study compared anti-HCV detection using an immunochromatographic strip test (fingerstick blood sample) with the standard commercial chemiluminescent microparticle immunoassay (Abbott ARCHITECT) on venous blood samples. A total of 4769 individuals from primary healthcare facilities in Phetchabun were tested. The RDT demonstrated a sensitivity of 88.7%, specificity of 99.6%, and accuracy of 98.9% [41]. Cases where the strip test yielded false negatives but the standard test was positive were often associated with undetectable or low viral loads. The study validated the feasibility of RDT-based screening in real-world field settings.

For individuals testing positive for anti-HCV via strip test, confirmatory testing was performed using qualitative HCV-RNA detection through real-time RT-PCR. This approach eliminated the need for quantitative HCV-RNA testing (viral load measurement). Patients with confirmed HCV-RNA positivity were immediately eligible for treatment with pan-genotypic DAAs, such as SOF/VEL, for 12 weeks. After completing treatment, SVR was assessed for 12 weeks post-treatment using qualitative HCV-RNA testing to confirm the cure.

**Figure 4:**
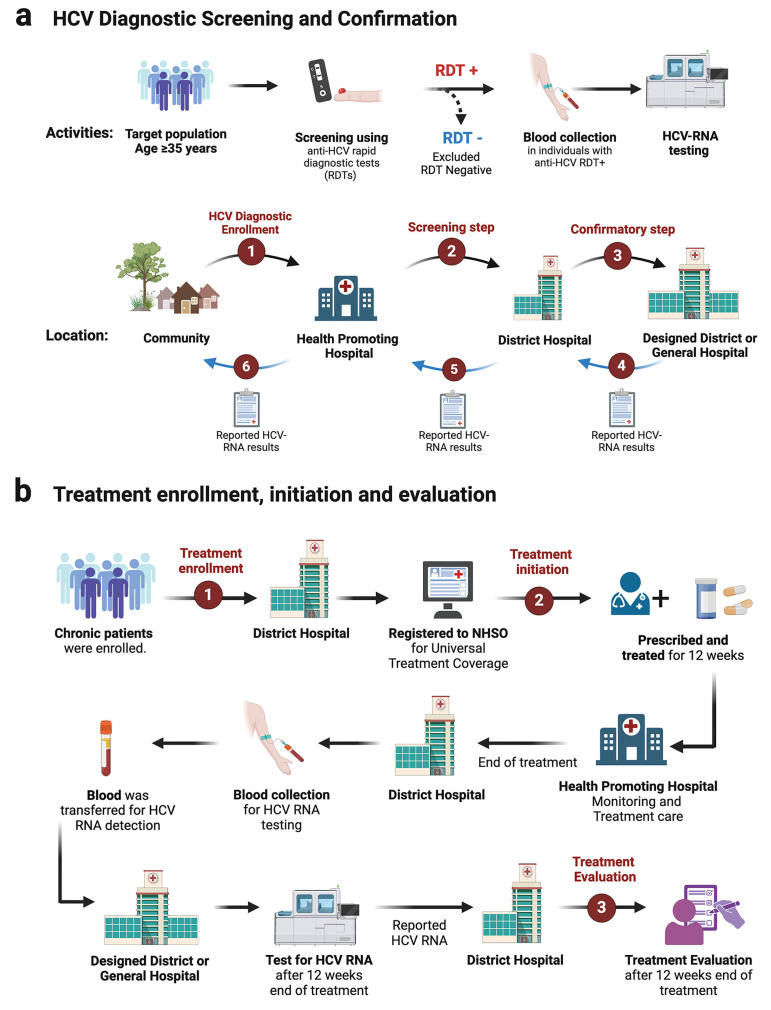
Diagram of the screening and treatment framework in the Phetchabun Model (test to treat). (**a**) Workflow and strategy for hepatitis C virus screening. (**b**) Treatment pathway and follow-up process for hepatitis C patients. RDT, rapid diagnostic test or strip test. (Created with BioRender.com).

Screening via strip test or RDT was conducted at the village level by well-trained nurses. Individuals testing positive were referred for confirmatory HCV-RNA qualitative testing at designated district hospitals in Phetchabun. The province established three HCV-RNA testing centers: one in Lom Sak (northern Phetchabun), one in Mueang Phetchabun (central Phetchabun), and one in Wichian Buri (southern Phetchabun). Once HCV-RNA results were obtained, they were relayed to district-level healthcare providers, where general practitioners initiated and monitored treatment. Upon treatment completion, final assessment and follow-up evaluations were conducted before sending blood samples to the designated HCV-RNA testing centers, as illustrated in the study framework (Figure 4a,b).

This model demonstrates a feasible and effective approach to HCV elimination, integrating decentralized rapid screening with streamlined confirmatory testing and treatment initiation, potentially serving as a blueprint for broader national implementation.

In the HCV microelimination study known as the Phetchabun Model, a province-wide approach was implemented using a simple and low-cost screening test priced at approximately USD 1 or less per test. Confirmation was performed using qualitative real-time RT-PCR, which is up to three times cheaper than the previously used quantitative test (viral load), costing only around USD 15 per test. Treatment involved the use of the generic drug Sof/Vel, which is affordable under the coverage of the NHSO. A cost–benefit analysis is planned for future study.

## 8. Microelimination: Phetchabun Implementation

**Figure 5:**
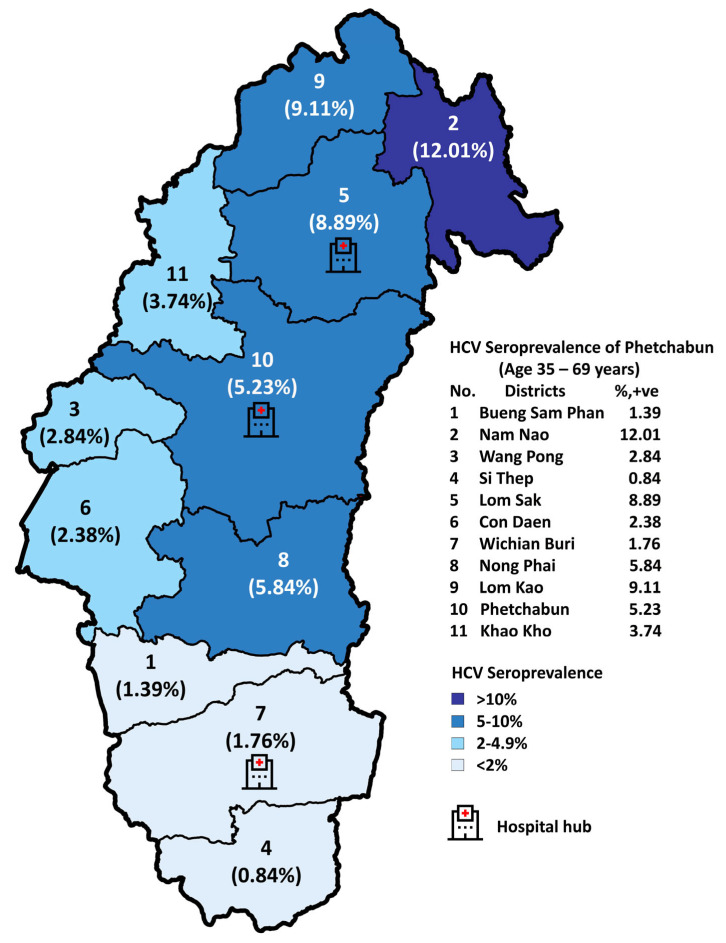
Seropositive rates of anti-HCV RDT across eleven districts in Phetchabun province from November 2019 to 7 February 2025.

For the implementation strategy in Phetchabun province, the test-to-treat plan under the Phetchabun Model was in operation from Nov-2019 to 7-Feb-2025. Screening was conducted in primary healthcare settings using a strip test, overseen by the district hospital. Confirmation was performed through qualitative real-time RT-PCR at three designated general hospital hubs. The screening targets individuals who were born before 1992, with a total population goal of 324,916 individuals. As of now, 288,003 individuals have been tested using strip tests or RDTs, accounting for 88.64% of the target population (Figure 5). Among those tested, 14,054 individuals (4.88%) were positive for anti-HCV strip tests and subsequently underwent qualitative HCV-RNA testing using real-time RT-PCR. A total of 11,639 individuals were tested for HCV-RNA, and 8451 (72.61%) were confirmed positive. The details of detection rates across different districts and the overall Phetchabun province results are presented in Table 3.

Treatment has been decentralized to the district level, with each district managing its treatment while evaluation and HCV-RNA testing are performed at designated centers. Among the 8451 HCV-RNA-positive cases, 167 cases are waiting for treatment and 328 individuals passed away while awaiting treatment, including 117 deaths from liver cancer and 151 from other causes. There were 336 cases with limitations for treatment. A total of 7452 individuals (88.18%) underwent standard 12-week treatment with DAA SOF/VEL under the NHSO program. Of these, 1170 patients received treatment outside of Phetchabun, while 6282 patients (84.29% of detected cases) were treated within the province.

**Figure 6:**
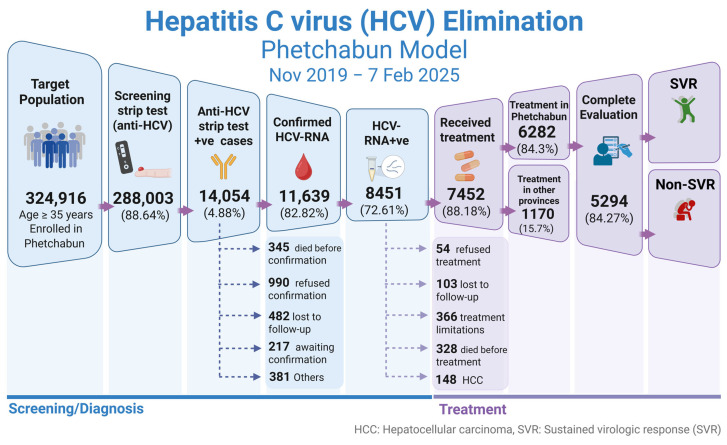
Progress in screening and treatment of hepatitis C in the population of Phetchabun province. The Phetchabun Model was implemented from Nov-2019 to 7-Feb-2025. SVR, sustained virological response. (Created with BioRender.com).

Among those who completed treatment and underwent evaluation (5294 individuals), SVR was achieved in 5084 cases (96.03%), while 210 cases (3.97%) remained HCV-RNA positive. The treatment outcomes are illustrated in Figure 6. Throughout the treatment process, patients received counseling and education on HCV transmission prevention, reinfection risks, and the dangers of substance abuse and alcohol consumption, which could impact future liver disease outcomes.

In 2023, the NHSO of Thailand officially implemented guidelines for the screening and treatment of hepatitis B and C infections [40]. Under these regulations, individuals born before 1992 are eligible for screening and, if diagnosed, are enrolled in the designated treatment program. HCV core antigen (HCVcAg) testing exhibits high sensitivity and provides results comparable to HCV-RNA testing [42,43]. Consequently, the NHSO has approved the use of HCVcAg for treatment decisions [44,45]. Patients who test positive for HCVcAg are eligible to proceed with treatment. Screening for hepatitis B and C is conducted using a strip test with a fingertip blood sample. Those who test positive undergo confirmatory testing for active infection using either the HCV core antigen test or HCV-RNA detection through qualitative or quantitative methods, without a predetermined viral load threshold [44,45]. For treatment, pan-genotypic DAAs, specifically SOF/VEL, are provided [44,45,46]. The eligibility criteria for healthcare providers administering treatment have also been revised [44,45,46]. Previously, only gastroenterologists or internal medicine specialists with extensive training were authorized to treat patients. However, the updated policy now allows general practitioners who have completed specific training to provide treatment, significantly improving access to diagnosis and care. This initiative aims to enhance hepatitis elimination efforts by 2030. However, screening coverage remains limited in some provinces due to various challenges, particularly shortages in healthcare personnel [46].

## 9. Monitoring and Surveillance

Public awareness campaigns were integrated into the diagnostic process, with all data recorded within the NHSO system to compile national-level data [47,48]. This allows for the assessment of real-world treatment effectiveness and future implications, particularly in the context of microelimination in Phetchabun. To further evaluate outcomes and the impact of microelimination, periodic surveys will be conducted among different population groups, including testing for both anti-HCV and HCV-RNA in the province and through the national surveillance system [48].

## 10. Challenges and Barriers

Phetchabun experiences significant population movement, including both in-migration and out-migration, as well as an influx of migrant workers from neighboring countries. These dynamics continuously affect screening and treatment coverage. Individuals migrating into Phetchabun should undergo hepatitis screening, and those found positive should enter treatment to prevent further disease transmission [27].

Qualitative real-time RT-PCR testing requires complex equipment and technical expertise. However, the adoption of small-scale automated machines has facilitated testing at smaller district levels, improving access to HCV-RNA diagnostics.

Treatment has become more accessible in recent years, thanks to its strong safety profile, which refers to a high level of safety and minimal side effects associated with HCV prevention measures. These include affordable generic DAAs, needle exchange programs, safe injection practices, screening and early diagnosis, and hygiene and medical safety protocols. Currently, the standard treatment lasts 12 weeks. However, future advancements may bring equally or more effective medications that could reduce the duration to 8 weeks or less for non-cirrhotic patients, improving adherence [49,50,51,52,53].

Patients who achieve SVR should undergo regular monitoring for potential risks and complications, particularly those with advanced fibrosis or cirrhosis. However, patients without advanced fibrosis who achieve SVR and have additional risk factors for HCC may be discharged. Monitoring should include assessing complications, such as portal hypertension and the risk of HCC, through periodic fibrosis evaluation, liver function tests, and tumor markers [54]. Additionally, surveillance for reinfection is essential. Patient education and counseling play a key role in raising awareness about infection prevention. In high-risk groups, repeat testing may be necessary to detect reinfection early, allowing for prompt treatment and preventing further transmission.

A crucial concern is the nearly 4% treatment failure rate, with patients remaining HCV-RNA positive after the 12-week treatment course. These cases require special attention to identify factors contributing to treatment failure, such as poor medication adherence, reinfection, or drug-resistant HCV strains. In cases where treatment with SOF/VEL fails to achieve a sustained virologic response (non-SVR), retreatment is necessary. This method involves extending the treatment duration to 12 weeks with SOF/VEL/Voxilaprevir (VOX) [55,56] or modifying the regimen using a triple therapy approach for 24 weeks with SOF/VEL + RBV in resource-limited settings [57]. Further studies are needed to optimize treatment strategies and improve therapeutic efficacy in such cases.

Continuous education on HCV infection, transmission, and its potential consequences remains essential, particularly for new at-risk populations. Although HCV is a severe infectious disease, stigma and discrimination must be minimized. Regular risk assessments and periodic surveys among high-risk groups will be crucial to achieve near-total elimination of hepatitis C by 2030.

## 11. Success Stories and Lessons Learned

The Phetchabun microelimination model, implemented over the past five years, has provided valuable insights into the test-to-treat approach for hepatitis C. The data generated can serve as a framework for expansion to other provinces in Thailand and as a model for LMICs to adapt their hepatitis C elimination strategies.

Thailand has the potential to scale up HCV screening and treatment nationwide. Strengthening primary healthcare systems will be critical in ensuring successful implementation. At the policy level, sustained commitment and continuous action are required, along with research initiatives to support sustained elimination efforts.

## 12. Limitations of This Study

The limitation of this study is that it is a narrative review relying primarily on data and content from Thailand, covering the past to the present. The aim is to provide a framework that can be applied to the elimination of hepatitis C. Therefore, it was not possible to analyze the data in the format of a systematic review or meta-analysis [58,59]. Additionally, the Thai-centric dataset may limit generalizability to regions with different health infrastructure, funding levels, or hepatitis C genotype distribution.

## 13. Conclusions

Hepatitis C remains a significant public health challenge in Thailand. However, with effective medications and treatment strategies now available, elimination is achievable. The Phetchabun microelimination model, which integrates disease burden assessment, baseline data collection, and a streamlined test-to-treat approach, demonstrates the feasibility of eliminating HCV. The screening process employs simple diagnostics, such as strip tests from finger-prick blood samples. Confirmatory testing with high-sensitivity qualitative HCV-RNA assays ensures accurate diagnosis. Standard pan-genotypic DAAs are used for treatment, with SVR assessments conducted post-treatment. For the small proportion of patients who do not achieve SVR, alternative treatment strategies must be considered. The Phetchabun Model illustrates that near-elimination of HCV is possible and can be expanded nationwide. Moreover, it serves as a valuable blueprint for other LMICs aiming to achieve the WHO’s goal of hepatitis C elimination by 2030.

## Figures and Tables

**Table 1:** Service coverage is required to achieve the WHO’s goal of eliminating HCV by 2030.

Topics	Descriptives	Targets
Prevention	Blood safety: Screening of all donated blood and blood products	100%
	Injection safety: Use of engineered devices	90%
	Harm reduction (distribution of sterile syringes/needles)	300 sets/years/PWID
Treatment	Diagnosis of HCV patients	90%
	Treatment of HCV patients	80%
Impact on leading elimination	Reduction in new HCV infections	90%
	Reduction in HCV-associated death	65%

PWID = person who injects drugs. The table was created and modified from [17].

**Table 2:** Estimation of HCV seroprevalence and disease burden of HCV infection in high-risk and general populations in Thailand.

Descriptives	PLHIV	PWID	MHD	Prison Inmates	High-Risk Sex Behavior	MSM	General Population
Number of datasets included	16	14	3	1	5	2	1
Number of study participants (n)	8388	8052	5073	1648	1150	1111	6069
Weighted pooled estimate of HCV seroprevalence (%)	13.45	58.86	13.13	5.22	2.00	1.62	0.56
95% CI of seroprevalence	9.41–17.50	39.30–78.42	2–23.86	4.25–6.40	0.55–7.00	0.88–2.36	-
Population at risk (n)	480,000	71,000	43,023	373,169	144,000	527,900	64,762,276
Estimated burden of HCV (n)	64,560	41,791	5649	19,480	2880	8552	363,475
Lower limit of burden	45,468	27,903	1033	15,860	792	4646	-
Upper limit of burden	84,000	55,678	10,265	23,883	10,080	12,458	-

Total estimated burden of HCV = 506,387. PLHIV = people living with HIV; PWID = people who inject drugs; MHD = patients undergoing maintenance hemodialysis; MSM = men having sex with men. The table was established based on data from reference [25].

**Table 3:** Prevalence of anti-HCV and HCV-RNA of the general population after implementing a simplified test-to-treat strategy, the Phetchabun Model, Nov-2019 until 7-Feb-2025.

Districts	Targets (n)	Screening of Total Cases (n)	Screening (%)	Anti-HCV RDT		HCV-RNA		
				Positive Cases (n)	Positive (%)	Cases Undergoing Confirmation (n)	Cases Undergoing Confirmation (n)	Positive (%)
Bueng Sam Phan	19,805	25,471	128.61	355	1.39	305	289	94.75
Nam Nao	5988	7512	125.45	902	12.01	869	529	60.87
Wang Pong	11,207	10,532	93.98	299	2.84	251	199	79.28
Si Thep	21,165	22,554	106.56	190	0.84	172	147	85.47
Lom Sak	55,641	50,419	90.61	4483	8.89	4039	3394	84.03
Chon Daen	21,929	20,065	91.5	477	2.38	303	177	58.42
Wichian Buri	44,226	41,931	94.81	739	1.76	701	464	66.19
Nong Phai	33,745	28,193	83.55	1646	5.84	1037	823	79.36
Lom Kao	27,580	21,617	78.38	1966	9.11	1829	1189	65
Phetchabun	2227	51,224	70.92	2680	5.231	1959	1102	56.25
Khao Kho	11,403	8485	74.41	317	3.74	174	138	79.31
Total	324,916	288,003	88.64	14,054	4.88	11,639	8451	72.61
